# The Experience of Working Family Caregivers: Implications for a National Paid Leave Policy

**DOI:** 10.1093/geroni/igaa057.2550

**Published:** 2020-12-16

**Authors:** Tiffany Washington, Lynn Friss Feinberg

## Abstract

Family caregivers provide the bulk of unpaid care to older adults. The typical family caregiver is a 49-year-old woman who works full time and simultaneously provides an average of 24 hours of care per week for an older relative. Unfortunately, their caregiving duties places them at risk for lost wages and termination due to frequent interruptions at work, especially in the absence of a national paid family leave policy. It is possible that such a policy could mitigate these risks; however, the United States is the only developed nation that lacks a national paid family leave policy for all workers. This symposium will highlight the psychosocial, economic, and health issues experienced by working caregivers, and conclude by linking presenters’ findings to implications for a national paid leave policy. To start, presenter one will describe findings from a scoping review on workplace experiences of female family caregivers. Next, presenter two will describe findings from a systematic review to explore predictors of the adoption and implementation of state-level paid family leave policies. Presenter three’s study examines interest in supportive services among working and non-working Black caregivers in the Deep South. Presenter four will describe factors associated with healthcare utilization of working caregivers using data from the Regional Healthcare Partnership – Region 17 Health Assessment Survey. The final presenter, HHS Advisory Council to Support Grandparents Raising Grandchildren co-chair, will describe development of policy initiatives to identify, coordinate, and promote information, resources, and best practices for working grandparents raising grandchildren.

